# Hepatic cysts: a survival guide

**DOI:** 10.1590/0100-3984.2024.0101-en

**Published:** 2025-04-25

**Authors:** Matheus Menezes Gomes, Gabriella Aquino Gouveia Cagliari, Eduardo Oliveira Pacheco, Ulysses Santos Torres, Giuseppe D’Ippolito

**Affiliations:** 1 Escola Paulista de Medicina da Universidade Federal de São Paulo (EPM-Unifesp), São Paulo, SP, Brazil

**Keywords:** Liver, Cystadenoma, mucinous, Echinococcosis, hepatic, Caroli disease, Liver abscess, Fígado, Cistadenoma mucinoso, Equinococose hepática, Doença de Caroli, Abscesso hepático

## Abstract

Hepatic cysts are quite common in the daily practice of radiologists and are generally described as simple cysts or as cystic lesions sparsely distributed throughout the parenchyma, often without the discrimination they merit. Simple cysts have, by definition, thin walls, one or two thin septa, and homogeneous fluid content. Such cysts include congenital epithelial cysts, biliary hamartomas, and peribiliary cysts, as well as those representing Caroli’s disease or polycystic liver disease. Complex cysts have variable walls, septa, and contents. They also have various etiologies. A detailed assessment of the clinical history and imaging characteristics can assist in making the diagnosis and choosing a course of clinical management. In this review, hepatic cysts are divided, for educational purposes, into five categories: congenital, traumatic, neoplastic, inflammatory, and miscellaneous.

## Abstract (pt)

Os cistos hepáticos são muito comuns na prática diária do radiologista e costumam ser descritos como cistos simples ou formações císticas esparsas pelo parênquima, muitas vezes sem as merecidas discriminações. Os cistos simples têm, por definição, paredes finas, até dois finos septos e conteúdo fluido e homogêneo, e englobam os cistos epiteliais congênitos, os hamartomas biliares, os cistos peribiliares, a doença de Caroli e a doença hepática policística. Os cistos complexos, por sua vez, têm paredes, septos e conteúdo variáveis, podendo ter diversas etiologias, e uma avaliação detalhada da história clínica e das características de imagem pode auxiliar o diagnóstico e o manejo clínico. Neste artigo os cistos hepáticos foram classificados, para fins didáticos, em cinco categorias: congênitos, traumáticos, neoplásicos, inflamatórios e miscelânea.

## INTRODUCTION

Hepatic cysts are quite common in the daily practice of radiologists. However, unlike cysts in other organs, such as the kidneys and ovaries, for which there are well-defined protocols for description, radiological classification, and diagnostic workup^(1^, ^2)^, those in the liver are usually reported as simple cysts or as sparse cystic formations throughout the parenchyma, often without the discrimination they merit. Cysts can be classified as simple or complex. In the various imaging methods, a simple cyst presents with a homogeneous liquid component—anechoic on ultrasound, with density close to zero on computed tomography (CT), and hypointense on T1-weighted magnetic resonance imaging (MRI) and markedly hyperintense on T2-weighted images—with thin walls, no vegetation or calcifications and no enhancement or noticeable flow. A complex cyst is one that does not have all of those characteristics^(3)^. In general, unless they are symptomatic, simple cysts do not merit concern, monitoring, or intervention. In contrast, complex cysts can require procedures for diagnostic clarification and treatment^(3)^.

The differential diagnosis of a hepatic cyst includes benign lesions (congenital, infectious/inflammatory, orc traumatic) and malignant lesions (e.g., biliary cystic neoplasms, cystic metastases, and cystic hepatocellular carcinoma), and a more careful evaluation could reveal signs of aggressiveness of the cyst in question and could inform decisions regarding specific management.

In this review article, we present the typical and atypical imaging findings of the various hepatic cysts, highlighting the aspects that allow a more specific diagnosis, and these lesions will be divided, for didactic purposes, into five categories (Table 1): congenital, traumatic, neoplastic, inflammatory, and miscellaneous.

**Table 1:** Summary of the main categories and main findings of the various liver cysts.

Category	Type
**Congenital**	Simple	Single or multiple; thin-walled; homogeneous; T2 hyperintensity
	Caroli’s	Central dot sign; communication with the bile duct
	Hamartoma	Multiple, small (< 1.0 cm), and homogeneous
	Ciliated	Liver segment IV subcapsular; variable content
	Polycystic liver disease	Multiple and large; increased liver volume; with or without renal cysts
**Traumatic**	Intrahepatic biloma	Biliary trauma or manipulation; homogeneous; without septa
	Seroma	Fluid and homogeneous collection in surgical site
	Hematoma	Trauma; hyperdensity/T1 hyperintensity; look for active bleeding
**Neoplastic**	Mucinous cystadenoma	Middle-aged women; multilocular; fibrous capsule; calcifications
	Cystic metastasis	Extrahepatic malignancy; multiple lesions; ring enhancement
	Cystic hepatocellular carcinoma	Cirrhosis; previous embolization; hypervascular areas; pseudocapsule
	Undifferentiated sarcoma	Children and young adults; rare; large cyst-like mass; multiple septa
**Inflammatory**	Pyogenic abscess	Clinical signs of infection; parietal enhancement; double-target sign; gas; perfusion disturbance
	Hydatid cyst	Positive serology; daughter cysts; honeycomb appearance; calcifications
	Amebic abscess	Endemic area; unilocular and peripheral collection (+ right liver lobe); “target” appearance; diaphragmatic rupture
	Fungal microabscess	Immunocompromised patients; multiple, small, and hypodense
**Miscellaneous**	Peribiliary cyst	Multiple and small, with peribiliary distribution
	Giant hemangioma with air–fluid level	Lesion > 5 cm; areas of liquefaction with an air–fluid level

## CONGENITAL CYSTS

### Simple epithelial cysts

**Figure 1:**
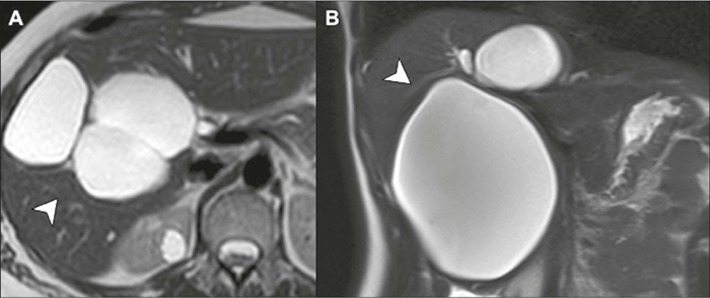
Simple epithelial cysts. T2-weighted MRI in the axial and coronal planes (**A** and **B,** respectively), showing thin-walled cysts, with a homogeneous fluid content, which can be unilocular or contain up to two thin internal septa (arrows).

Simple epithelial cysts are cysts with thin, regular walls, which can have a few thin septa and are composed of cuboidal epithelium. Their size varies, ranging from very small to 30 cm in diameter, and they are filled with homogeneous fluid content (Figure 1).

Conditions other than isolated congenital cysts, such as biliary hamartomas and polycystic liver disease, can fall into the category of simple epithelial cysts, despite presenting distinct pathophysiologies^(4)^.

### Caroli’s disease

Caroli’s disease is a rare, congenital, autosomal recessive condition resulting from a malformation of the ductal plate and abnormal intrahepatic bile duct development, which can be accompanied by congenital hepatic fibrosis and medullary sponge kidney (renal tubular ectasia), in which case it is known as Caroli’s syndrome^(5)^. In Caroli’s disease, there is an estimated 7% risk of developing cholangiocarcinoma^(4)^.

**Figure 2:**
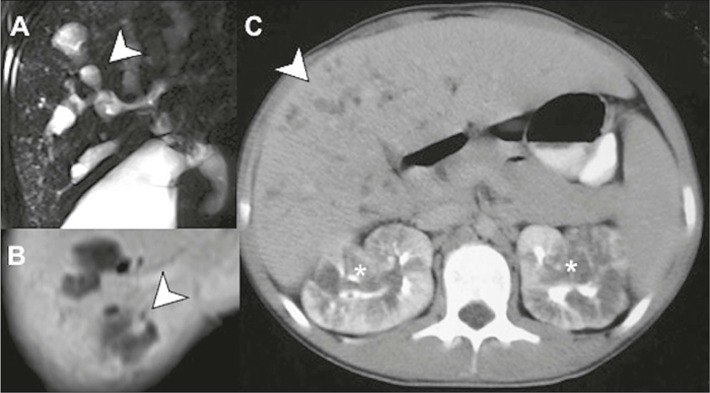
Caroli’s disease. MRI cholangiography with maximum intensity projection **(A)** showing multifocal cystic dilatations of the intrahepatic bile ducts (arrow). Contrast-enhanced T1-weighted sequence **(B)** showing the central dot sign, representing a portal branch in the middle of the dilated bile duct (arrow), which is quite characteristic of this condition. **C:** Contrast-enhanced CT scan of a patient with Caroli’s disease, in the pyelographic phase, showing dilatation of the biliary tree (arrow) and medullary sponge kidneys (asterisks).

Because the cystic dilatations of the bile ducts in Caroli’s disease communicate with the biliary tree, they tend to retain contrast in the late phases of MRI studies with hepatobiliary-specific contrast medium. Fibrovascular bundles can be detected within the outpouchings, configuring the central dot sign, a finding relatively specific to these lesions (Figure 2). They are categorized as Todani type V, a classification directed at choledochal or biliary cysts^(4)^.

### Ciliated hepatic foregut cyst

Ciliated hepatic foregut cysts are rare cysts that have a predilection for males. Although their histogenesis is uncertain, some authors have suggested that they originate from the embryonic foregut, with a histological constitution similar to that of a bronchogenic cyst, and are thus formed by pseudostratified ciliated columnar epithelium^(6)^. Although there have been a few reports of malignant degeneration to the squamous cell carcinoma subtype^(7)^, such cysts are generally asymptomatic and benign, therefore not requiring resection.

**Figure 3:**
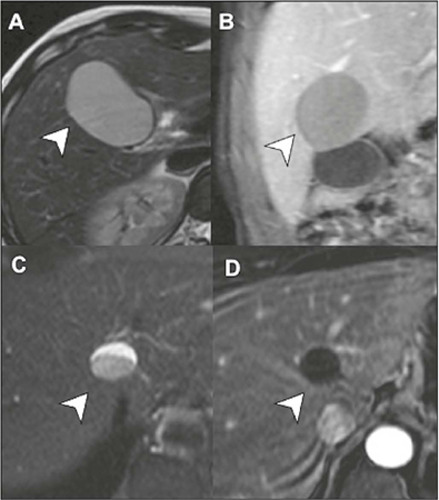
Ciliated cysts on MRI. Contrast-enhanced T2-weighted images, in the axial and coronal planes **(A** and **B,** respectively), showing a unilocular cyst near the posterior capsule of liver segment IV (arrows) with slightly thick contents and no solid components. In another patient, a cyst in a similar location is seen on an axial T2-weighted image **(C),** in this case with a air-fluid level (arrow), also without detectable enhancement on a contrast-enhanced T1-weighted image with digital subtraction **(D).**

On imaging examinations, ciliated cysts appear as solitary, typically unilocular, lesions, generally smaller than 3.0 cm, predominantly located along the capsular surface of liver segment IV. On ultrasound, CT, and MRI, they have dense content, because they can contain mucin, high levels of proteins, or even lipid material^(6)^, as illustrated in Figure 3.

### Biliary hamartomas

**Figure 4:**
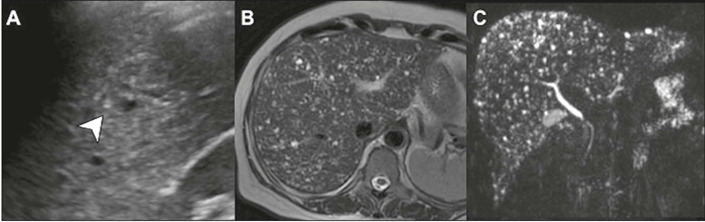
Biliary hamartomas. Ultrasound **(A)** showing small cysts with the typical comet-tail artifact (arrow). T2-weighted MRI sequence **(B)** and MRI cholangiography **(C)** clearly showing multiple small hyperintense cysts distributed throughout the liver parenchyma.

Biliary hamartomas, also known as Von Meyenburg complexes, result from benign changes in the development of the bile ducts, included in the spectrum of ductal plate malformation, and give rise to multiple small hepatic cysts. Despite their origin, these cysts have a unique histological architecture and do not communicate with the bile ducts. Like ciliated cysts, these lesions are asymptomatic and have a benign course. Imaging examinations typically reveal multiple cysts smaller than 1.0 cm distributed throughout the liver parenchyma, and each of those cysts usually exhibit a comet-tail artifact on ultrasound and show discrete peripheral enhancement on axial images^(8)^, as depicted in Figure 4.

### Polycystic liver disease

**Figure 5:**
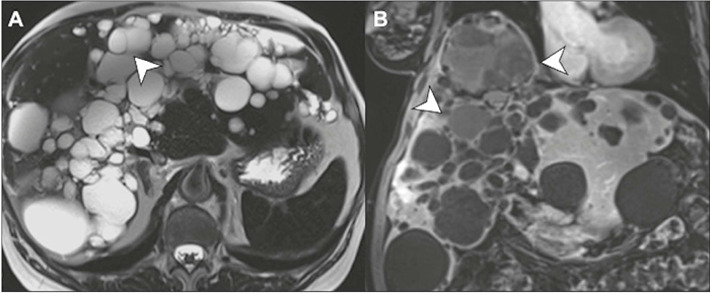
Polycystic liver disease on MRI. Note that, as with renal involvement, there is a tendency for liver volume to increase due to multiple, confluent cysts. On an axial T2-weighted image **(A),** most cysts appear as simple cysts, with thin or no internal septa (arrow). On a contrast-enhanced coronal T1-weighted image **(B),** the cysts can have variable signal intensity (arrows) due to the blood/high protein content, although they do not show nodular enhancement.

Polycystic liver disease is an uncommon condition that is within the spectrum of autosomal dominant polycystic kidney disease and is related to malformation of the ductal plate. It is characterized by multiple hepatic cysts, usually more than 20, although as few as four cysts can suggest the diagnosis if there is a family history of the disease. The cysts usually have a simple appearance on imaging, being mostly unilocular and thin-walled, and can present variable density or signal if there is blood or high protein content^(9)^, as shown in Figure 5.

Patients with polycystic liver disease are rarely symptomatic, with symptoms being observed in only 3% of cases. When there are symptoms, including early satiety, gastroesophageal reflux, and even malnutrition in advanced cases, they typically occur because of liver expansion and the consequent compression of adjacent organs. In up to 45% of cases, the pancreatic cancer marker CA 19-9 is elevated, because of its increased production by the biliary epithelium of the cysts, although it has not been associated with malignancy^(10)^.

The treatment of polycystic liver disease tends to be conservative, including sclerotherapy, aspiration, and laparoscopic fenestration. Surgical treatment, which should be performed at specialized centers with hepatobiliary surgeons and multidisciplinary discussions, includes everything from hepatectomy (in selected cases) to liver transplantation, the latter being indicated when there is extensive liver involvement and high morbidity^(9)^.

### Traumatic cysts

#### Biloma

**Figure 6:**
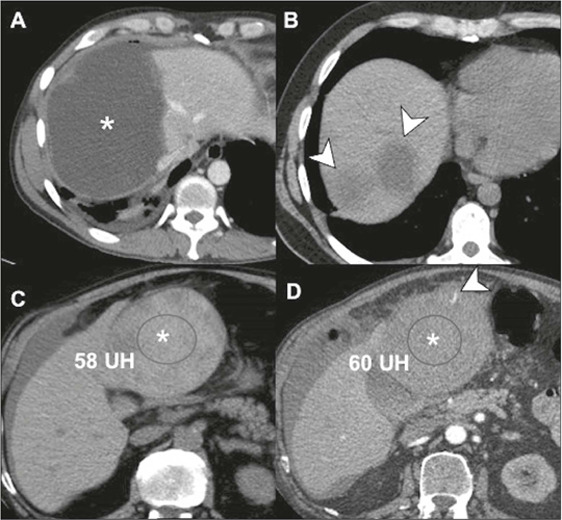
CT scans of cysts of traumatic origin. **A:** Biloma. A large pericapsular collection (asterisk) is observed in the manipulated region of the hepatic dome. Note the heterogeneous content, with some gaseous foci, and the thick walls with enhancement, denoting an inflammatory component. **B:** Seromas at nodulectomy sites. Note the small, hypodense, homogeneous fluid collections (arrows), a relatively common finding in the postoperative context. **C,D:** Hematoma. A hyperdense collection without significant enhancement can be seen in the left liver lobe (asterisk), containing a small focus of active bleeding in the arterial phase (arrow).

A biloma is defined as an abnormal collection of ex-traductal bile, usually related to iatrogenic interruption of the biliary tract during procedures such as biliary surgery, endoscopic retrograde cholangiopancreatography, and transarterial embolization. The extravasation of bile, usually at a low rate of flow, causes an inflammatory reaction in the surrounding tissue, resulting in fibrosis and encapsulation of fluid, which usually occurs near the surface of the liver at the manipulation site. Bilomas present as unilocular collections, with content that is hypodense (< 20 HU) on CT and markedly hyperintense on T2-weighted MRI, and can have thin or slightly thickened walls that show contrast enhancement (Figure 6). The presence of multiple septa with intense enhancement, restricted diffusion (due to dense content), and infiltration of the adjacent fat should raise the suspicion of a superimposed infection, within an appropriate clinical and laboratory context. On MRI with hepatobiliary-specific contrast, a biliary fistula might be seen in the delayed phase^(11)^.

#### Seroma

Seromas are predominantly lymphatic collections that are common in the context of trauma, particularly in the postoperative period. On axial imaging, they appear as of homogeneous fluid collections with no enhancement or minimal peripheral enhancement (Figure 6). Seromas are common and typically resolve spontaneously; drainage is reserved for specific cases, such as those of patients with seromas that are very large or are infected^(12)^.

#### Hepatic hematoma

The liver, like the spleen, is one of the organs most commonly affected in blunt abdominal trauma, which can result in the formation of subcapsular or intraparenchymal hematomas. Although acute hematomas are usually hyperdense (40–60 HU) on unenhanced CT, the use of contrast medium is recommended, not only for the detection of lacerations and hematomas that are isodense to the parenchyma but also for the investigation of active bleeding, which may require an interventional or surgical approach^(13)^, as illustrated in Figure 6.

### Neoplastic cysts

#### Mucinous cystic neoplasms

**Figure 7:**
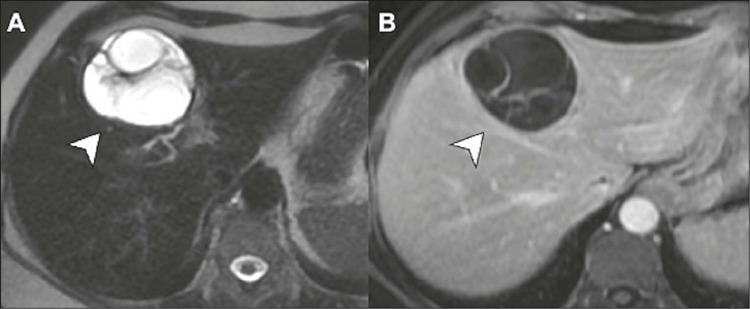
Mucinous cystadenoma in a 56-year-old female patient. **A:** Axial T2-weighted MRI showing a multiloculated cyst with thick walls and septa in liver segment IVa (arrow). **B:** Axial Tl-weighted MRI with contrast enhancement of walls and septa, without evident mural nodules.

Previously known as biliary cystadenomas, mucinous cystic neoplasms account for less than 5% of all cystic liver lesions and are most common in middle-aged women. These lesions are lined with mucin-producing columnar epithelium overlying an ovarian-like stroma and do not normally communicate with the bile ducts^(14)^. They can be asymptomatic; when present, the symptoms are non-specific, including abdominal pain and an early sensation of satiety, depending on the cyst volume. Their size can increase after oral contraceptive use and during pregnancy, suggesting a hormonal influence. Elevated levels of tumor markers such as CEA and CA19-9 can inform decisions regarding management and control, although normal levels do not exclude invasive carcinoma. Some imaging findings that help differentiate mucinous cystic neoplasms from simple cysts are the following (presented by the former): predilection for the left liver lobe; dilatation of the bile ducts upstream of the lesion; no retractions of the cyst walls; and alterations in the surrounding hepatic parenchyma. Other imaging findings of mucinous cystic neoplasms include a multiloculated appearance (Figure 7), calcifications, mural nodules, and irregular walls, the last two findings being suggestive of malignancy^(15)^.

#### Cystic metastases

**Figure 8:**
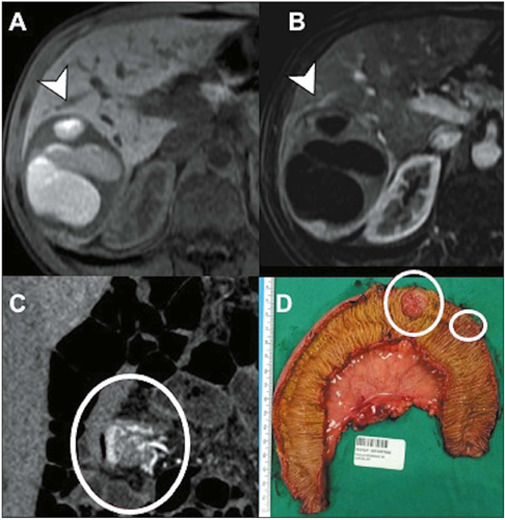
Liver metastasis from a neu-roendocrine tumor of the small bowel. Axial T1-weighted images before and after contrast injection **(A** and **B,** respectively), showing a predominantly liquid lesion in the right liver lobe, with blood content and coarse peripheral enhancement (arrows). Coronal CT reconstruction **(C)** showing the primary hypervascular lesion in the small bowel (circle) and its representation in the surgical specimen **(D).**

**Figure 9:**
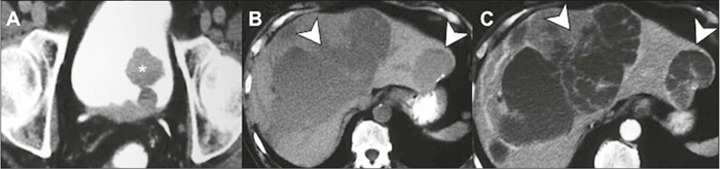
Liver metastasis from urothelial carcinoma of the bladder. Axial CT scan in the pyelographic phase, showing the primary bladder lesion **(A)** and a rare pattern of cyst-like liver metastases **(B,C),** with enhancement mainly restricted to their walls and with irregular septa (arrows).

Some neoplasms can generate cyst-like liver metastases because of the development of marked intratumoral necrosis or even due to the predominantly liquid content of the primary lesions, such as mucinous ovarian or pancreatic neoplasms, as well as neuroendocrine tumors (Figure 8). In hypervascular tumors with rapid growth and insufficient blood supply, such as metastases from gastrointestinal stromal tumor, sarcoma, melanoma, or angio-sarcoma, marked necrosis tends to appear. Other potential primary sites for cystic metastases include colorectal adenocarcinoma, squamous cell carcinoma of the lung, sarcomas, and melanomas. These lesions can have some characteristics that indicate their aggressiveness, such as irregular walls or septa (Figure 9), with enhancement or a rapid growth rate. However, in some cases, complete necrosis or degeneration generates an appearance similar to that of a simple hepatic cyst, making the initial diagnosis of cystic metastasis difficult^(16)^. Therefore, comparison with previous examinations can be quite useful for differentiating between a cystic metastasis and a simple cyst.

#### Cystic hepatocellular carcinoma

**Figure 10:**
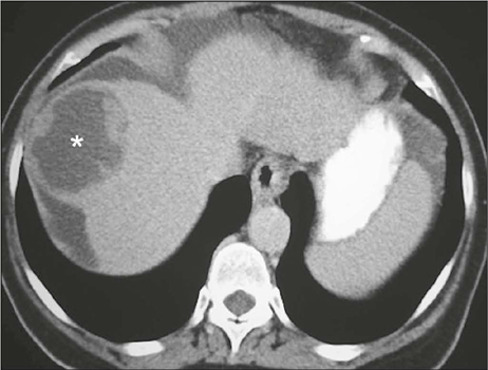
Cystic hepatocellular carcinoma with necrosis/cystic degeneration (asterisk), confirmed by CT-directed percutaneous biopsy.

Cystic degeneration is a rare presentation form of hepatocellular carcinoma. Although its pathogenesis has not been well elucidated, several mechanisms have been proposed, including arterial thrombosis, inflammation, rapid tumor growth, and androgen therapy^(17)^. Many of the reported cases of cystic hepatocellular carcinoma consist of single, multilocular lesions in a non-cirrhotic liver, which makes its diagnosis even more challenging. Nevertheless, small solid components with arterial hyperenhancement and lavage between the cysts should be actively sought when there is clinical suspicion, especially in cirrhotic livers with elevated alpha-fetoprotein^(18)^, as shown in Figure 10.

#### Undifferentiated embryonal sarcoma

**Figure 11:**
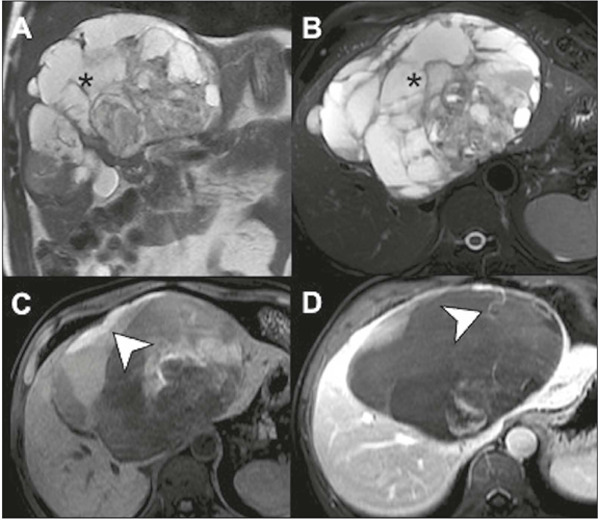
Undifferentiated sarcoma in a nine-year-old child. **A:** MRI showing a large heterogeneous mass (asterisk) with marked necrosis, characterized by a predominance of signal hyperintensity on T2-weighted images **(A,B),** sometimes forming air-fluid levels, together with layers of blood content (arrow in **C),** as well as predominantly septal and peripheral enhancement (arrow in **D).**

An undifferentiated embryonal sarcoma is a rare, highly aggressive primary liver neoplasm that occurs predominantly in children, usually between 6 and 10 years of age, but is also found in adults. Unlike hepatoblastoma and hepatocellular carcinoma, serum alpha-fetoprotein levels are not elevated in cases of undifferentiated embryonal sarcoma. In such cases, there can be constitutional symptoms and increased abdominal volume. On CT and MRI, the characteristics of an undifferentiated embryonal sarcoma can resemble those of cystic lesions, probably because of the high water content of its myxoid stroma (Figure 11). However, that myxoid component usually shows contrast enhancement and has a heterogeneous appearance, which differentiates it from a simple cyst. Although it has a poor prognosis, with a reported median survival of less than one year, multiagent chemotherapy followed by resection of the lesion has been shown to be a relatively effective treatment^(19)^.

### Inflammatory cysts

#### Pyogenic abscess

Most pyogenic liver abscesses are polymicrobial, with the main reported agents being *Klebsiella pneumoniae* and *Escherichia coli.* Their pathogenesis is multifactorial and can result from ascending cholangitis, hematogenous spread of a gastrointestinal infection, or direct inoculation from penetrating trauma or an invasive procedure.

**Figure 12:**
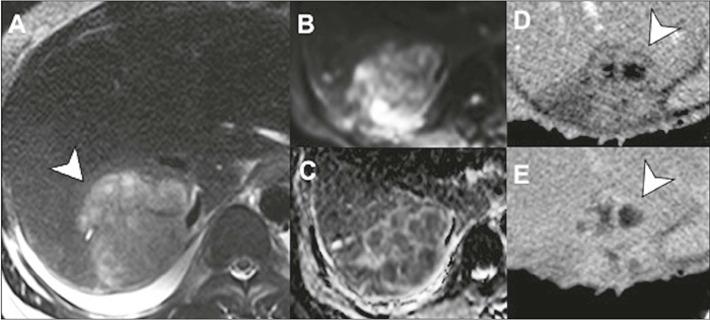
Pyogenic liver abscess in a patient with abdominal pain, fever, and leukocytosis. On MRI, the collection is seen to have thick walls and a hyperintense signal on T2-weighted images (arrow in **A),** in addition to a multiloculated appearance and content with markedly restricted diffusion **(B,C).** Contrast-enhanced CT of the same patient **(D,E)** showing the “double-target” sign, characterized by a hyperdense inner border surrounded by a hypodense ring (edema), the latter with delayed enhancement (arrows).

On CT, pyogenic liver abscesses appear as round, hypodense masses with well-defined margins and heterogeneous peripheral enhancement. They may manifest as a single, nonloculated collection of fluid, a multiloculated cystic mass, a solid-appearing (phlegmonous) area, or small multifocal lesions. Perfusion disturbance and parenchymal edema around the lesion are usually present. On MRI, marked restricted diffusion is typically seen due to the high protein content. Contrast-enhanced CT images show the “double-target” sign, which is characterized by a high-density inner rim (membrane) with early enhancement surrounded by a hypodense outer ring (edema) with late enhancement. (Figure 12). Although gas can be identified in up to 20% of liver abscesses, that finding should be interpreted with caution, because gas can be present in areas of necrosis, such as those developing after ablation of a liver tumor. Treatment includes image-guided drainage and prolonged antibiotic therapy, usually for four to six weeks^(20)^.

#### Hydatid cyst

**Figure 13:**
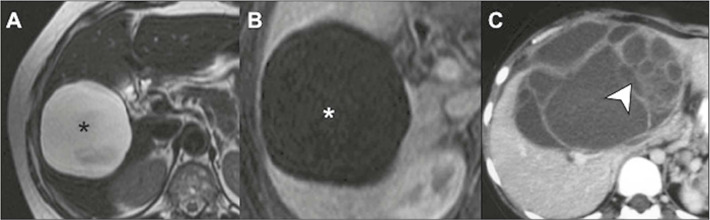
Types I and II hydatid cysts. On MRI, type I **(A,B)** presents as a thin-walled, unilocular lesion (asterisk) in the right liver lobe, resembling a simple epithelial cyst. Type II **(C)** has a multilocular appearance, with thin septa and confluent daughter cysts (arrow).

**Figure 14:**
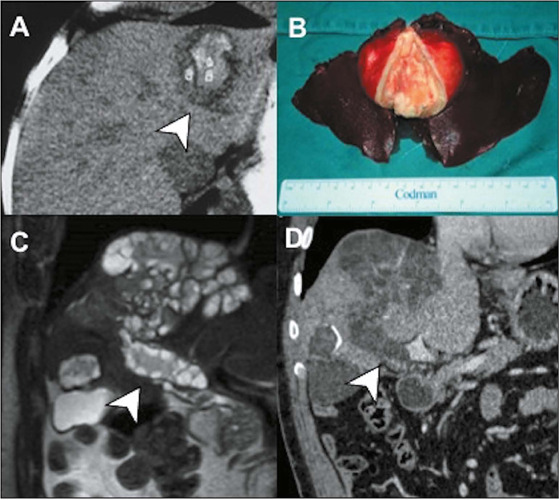
Types III and IV hydatid cysts. Unenhanced CT scan **(A)** showing a cyst with a diffusely calcified center (arrow) in the left liver lobe and its representation in the surgical specimen **(B),** indicative of an inactive (type III) hydatid cyst. Coronal T2-weighted MRI sequence **(C)** showing the focal rupture of a large hydatid cyst into the portal vein (arrow), a finding corroborated on a contrast-enhanced CT scan **(D).** This aspect represents angioinvasive hydatidosis, a rare type of presentation of hepatic cystic echinococcosis, classified as a type IV hydatid cyst.

Hepatic cystic echinococcosis, also known as hepatic hydatid cysts, is a parasitic infection caused by the larvae of a tapeworm of the genus *Echinococcus.* The disease is endemic in many parts of the world, especially in some countries in North America, South America, Asia, and Eastern Europe, as well as in Australia and New Zealand. The liver acts as the first line of defense and is therefore the organ most commonly affected by the disease. The disease is usually asymptomatic, although that can change depending on the stage of growth and mass effect of the cysts on adjacent organs, as well as secondary complications, such as rupture. On imaging, hydatid cysts preferentially affect the right liver lobe and can be single or multiple, with or without involvement of other organs. Hydatid cysts are divided into four types. Type I consists of a typically unilocular lesion with peripheral enhancement, which can have some thin septa, making a differential diagnosis with the simple epithelial cyst. Type II, the multivesicular type, appears as a cystic lesion with multiple septa or “daughter cysts” in a honeycomb pattern (Figure 13). In their most advanced stage, the cysts become partially or completely calcified (type III) and are considered inactive (Figure 14). Type IV represents cysts that have secondary complications, such as rupture, which occurs in 50–90% of cases, generally related to age, a chemical reaction or a defense mechanism (Figure 14). Another complication is superinfection, which has been reported to occur in up to 25% of ruptured hydatid cysts, in which the presence of gas, an air–fluid level, or communication with the bile ducts or other organs can suggest its diagnosis^(21)^.

#### Amebic abscess

**Figure 15:**
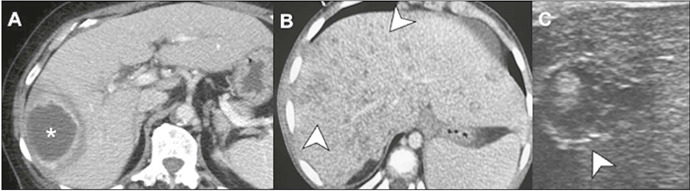
Specific liver infections. Contrast-enhanced CT scan **(A)** showing an amebic abscess presenting as a thick-walled, “target-shaped” collection (asterisk) in the right liver lobe. Contrast-enhanced CT scan **(B)** showing fungal microabscesses presenting as multiple hypodense lesions widely distributed throughout the liver parenchyma (arrows), simulating metastases. Ultrasound **(C)** showing a fungal microabscess with a central nidus and an external hyperechoic halo, characterizing the “bull’s eye” sign.

Amebic liver abscess is the most common extraintestinal complication of infection with *Entamoeba histolytica.* It is endemic in Africa and Southeast Asia, as well as in Central and South America. It predominantly affects men, and the most common clinical manifestations are right upper quadrant pain, fever, cough, and hepatomegaly. Diagnosis requires detection of *E. histolytica* antigen or DNA in stool samples and antibodies to *E. histolytica* in blood serum. Although amebic abscesses can be indistinguishable from pyogenic abscesses, some imaging features can suggest the diagnosis of amebic abscess. Such features include a single or unilocular collection (in 75% and 70% of patients with a amebic abscess, respectively), typically found in the right liver lobe, with or without a “target” or “double-rim” appearance (Figure 15). Amebic liver abscesses are usually located near the hepatic capsule, with extrahepatic extension being common; when located in the dome, they can be accompanied by diaphragmatic rupture, a sign considered highly suggestive of the diagnosis^(20)^. Treatment is typically with metronidazole, which is sufficient to eradicate the disease in most patients^(22)^, with drainage being reserved for refractory cases. However, amebic abscesses can take up to two years to resolve on imaging examinations, and the treatment is therefore guided by clinical parameters^(20)^.

#### Fungal microabscesses

Fungal infections of the liver, most commonly caused by *Candida* spp., usually develop after the translocation of fungi from the intestine to the liver via the portal circulation, creating microabscesses. The risk factors for such infection include an underlying malignancy, neutropenia, and immunosuppression. On ultrasound, the most common features are hypoechoic nodules (fibrosis) developing in an area of previous inflammation, the “wheel within a wheel” appearance, defined as a central hypoechoic area of necrosis surrounded by a hyperechoic zone of inflammatory cells, and the “bull’s eye” appearance, characterized by a central hyperechoic nidus surrounded by a hypoechoic halo^(23)^. On CT, fungal microabscesses usually appear as small, round, hypoattenuating lesions with a miliary pattern of distribution. The “wheel within a wheel” appearance might also be present (Figure 15). On MRI, the nodules are hyperintense on T2, with moderate enhancement after contrast ^(24)^. Although they can, at first glance, mimic metastases, the size and pattern of distribution can distinguish them from metastases, as can accompanying splenic involvement, which is common, as well as a clinical and laboratory picture suggestive of fungal infection.

### Miscellaneous

#### Peribiliary cysts

**Figure 16:**
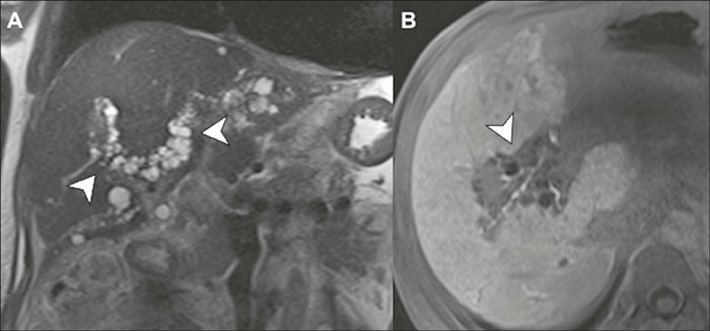
Peribiliary cysts. Coronal T2-weighted MRI scan **(A)** and contrast-enhanced axial Tl-weighted MRI in the hepatobiliary phase **(B)** showing multiple, small, simple-appearing cysts with a characteristic periductal distribution, which are often associated with chronic liver disease.

Peribiliary cysts represent cystic dilatation of glands in the periductal connective tissue and are usually found incidentally in patients with advanced chronic liver disease. Because they are lined by a single layer of columnar epithelium, these cysts have a simple appearance on imaging. In addition, they do not communicate with the bile ducts and have a characteristic peribiliary distribution that facilitates their diagnosis (Figure 16). Although they can, in rare cases, increase in size and cause obstruction, they are usually asymptomatic and have a benign course^(25)^.

#### Giant cavernous hemangioma with an air–fluid level

**Figure 17:**
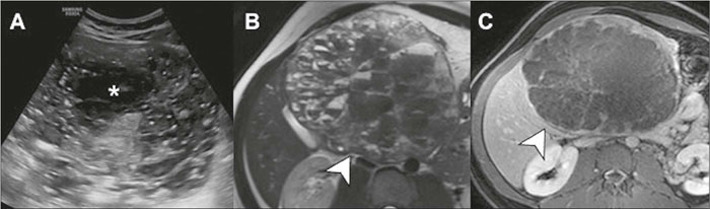
Giant cavernous hemangioma with an air-fluid level. A large, predominantly liquid mass can be seen in the left liver lobe. Note the multiloculated appearance with internal echoes (asterisk) on ultrasound **(A)** and multiple pockets with an air-fluid level on contrast-enhanced T2-weighted MRI **(B),** with predominantly peripheral enhancement **(C),** an atypical presentation pattern for hemangioma. The diagnosis was made by ultrasound-guided percutaneous biopsy.

Giant cavernous hemangiomas are a particular subtype of hepatic hemangiomas that, by definition, are larger than 5 cm. Although the radiological features of smaller cavernous hemangiomas may be present—marked T2 hyperintense signal and discontinuous peripheral nodular enhancement ^(26)^ —some of these lesions have extremely slow or stagnant blood flow, with sedimentation of red blood cells and consequent cystic appearance with formation of a fluid level. This aspect is represented by a lower layer that is hyperdense on CT and shows high signal intensity on T1-weighted MRI, with an upper layer consisting of fluid serum, which is hypodense on CT and has low signal intensity on T1-weighted MRI^(27)^, as shown in Figure 17.

## CONCLUSION

Hepatic cysts are frequently found on imaging studies, occurring in almost 15% of patients submitted to imaging of the pelvis or abdomen^(28)^. A wide variety of hepatic cysts of diverse etiologies can be encountered in radiology practice, and a detailed evaluation of the clinical history and imaging features, including location, number of lesions, enhancement pattern, and relationship to other structures, could indicate a specific etiology and thus guide individualized clinical management.
